# Genetic and Phenotypic Features of Schizophrenia in the UK Biobank

**DOI:** 10.1001/jamapsychiatry.2024.0200

**Published:** 2024-03-27

**Authors:** Sophie E. Legge, Antonio F. Pardiñas, Grace Woolway, Elliott Rees, Alastair G. Cardno, Valentina Escott-Price, Peter Holmans, George Kirov, Michael J. Owen, Michael C. O’Donovan, James T. R. Walters

**Affiliations:** 1 Centre for Neuropsychiatric Genetics and Genomics, Division of Psychological Medicine and Clinical Neurosciences, School of Medicine, Cardiff University, Cardiff, United Kingdom; 2 Leeds Institute of Health Sciences, Division of Psychological and Social Medicine, Faculty of Medicine and Health, University of Leeds, Leeds, United Kingdom

## Abstract

**Importance:**

Large-scale biobanks provide important opportunities for mental health research, but selection biases raise questions regarding the comparability of individuals with those in clinical research settings.

**Objective:**

To compare the genetic liability to psychiatric disorders in individuals with schizophrenia in the UK Biobank with individuals in the Psychiatric Genomics Consortium (PGC) and to compare genetic liability and phenotypic features with participants recruited from clinical settings.

**Design, Setting, and Participants:**

This cross-sectional study included participants from the population-based UK Biobank and schizophrenia samples recruited from clinical settings (CLOZUK, CardiffCOGS, Cardiff F-Series, and Cardiff Affected Sib-Pairs). Data were collected between January 1993 and July 2021. Data analysis was conducted between July 2021 and June 2023.

**Main Outcomes and Measures:**

A genome-wide association study of UK Biobank schizophrenia case-control status was conducted, and the results were compared with those from the PGC via genetic correlations. To test for differences with the clinical samples, polygenic risk scores (PRS) were calculated for schizophrenia, bipolar disorder, depression, and intelligence using PRS-CS. PRS and phenotypic comparisons were conducted using pairwise logistic regressions. The proportions of individuals with copy number variants associated with schizophrenia were compared using Firth logistic regression.

**Results:**

The sample of 517 375 participants included 1438 UK Biobank participants with schizophrenia (550 [38.2%] female; mean [SD] age, 54.7 [8.3] years), 499 475 UK Biobank controls (271 884 [54.4%] female; mean [SD] age, 56.5 [8.1] years), and 4 schizophrenia research samples (4758 [28.9%] female; mean [SD] age, 38.2 [21.0] years). Liability to schizophrenia in UK Biobank was highly correlated with the latest genome-wide association study from the PGC (genetic correlation, 0.98; SE, 0.18) and showed the expected patterns of correlations with other psychiatric disorders. The schizophrenia PRS explained 6.8% of the variance in liability for schizophrenia case status in UK Biobank. UK Biobank participants with schizophrenia had significantly lower schizophrenia PRS than 3 of the clinically ascertained samples and significantly lower rates of schizophrenia-associated copy number variants than the CLOZUK sample. UK Biobank participants with schizophrenia had higher educational attainment and employment rates than the clinically ascertained schizophrenia samples, lower rates of smoking, and a later age of onset of psychosis.

**Conclusions and Relevance:**

Individuals with schizophrenia in the UK Biobank, and likely other volunteer-based biobanks, represent those less severely affected. Their inclusion in wider studies should enhance the representation of the full spectrum of illness severity.

## Key Points

**Question:**

How do individuals with a diagnosis of schizophrenia recruited in a large volunteer-based research resource (UK Biobank) differ from those in the Psychiatric Genomics Consortium (PGC) or those recruited from clinical settings?

**Findings:**

In this cross-sectional study including more than 517 000 individuals, liability to schizophrenia in the UK Biobank had a high genetic correlation with the PGC. Compared with 4 clinically ascertained schizophrenia samples, UK Biobank participants with schizophrenia had significantly lower schizophrenia genetic liability as indexed by polygenic risk score, lower rates of copy number variants, and fewer phenotypic features of poor outcome.

**Meaning:**

In this study, individuals with schizophrenia in the UK Biobank had features of less severe illness, which indicates that registries such as the UK Biobank can help to capture the full range of heterogeneity in schizophrenia research.

## Teaser

This cross-sectional study compares genetic liability to psychiatric disorders in individuals with schizophrenia in the UK Biobank with individuals in the Psychiatric Genomics Consortium and compares genetic liability and phenotypic features with participants recruited from clinical settings.

## Introduction

Large population-based volunteer biobanks are increasingly being used to study human disease. Millions of participants across the world from newly available biobanks will be made available for research within the next 5 years. However, these samples are known to be subject to ascertainment biases,^1^ in particular healthy volunteer bias. For example, of the 9.2 million people invited to participate in the UK Biobank, the 5.5% that participated are disproportionately female, socioeconomically advantaged, and White. They are also less likely to be obese or to smoke, report fewer health conditions, and have lower mortality rates.^2^ While ascertainment bias clearly affects prevalence estimates, it has been argued that it does not affect exposure-disease associations or scientific inference.^3,4^ However, studies have shown that these biases can change effect sizes in genetic association studies^5^ and impact downstream analyses,^1^ and new methods are being developed to detect biases and offset them.^6^

It is unclear how these selection biases coupled with differing methods of identifying and defining affected status, such as the use of self-report and electronic health records, influence the features of schizophrenia cohorts identified through large population-based samples and how such cohorts will differ from clinically ascertained samples. Nonrandom participation does not just affect population-based cohorts. Clinically ascertained studies of serious mental illness, typically through secondary care, can be underrepresented for those who have difficulty obtaining such care due to socioeconomic and other causes of health care disparities.^7,8^ Moreover, they are also likely to be underrepresented for people with mild forms of the disorder who may not be referred to secondary care, much less hospitalized, while those with excellent clinical outcomes are likely to be discharged early from secondary care, biasing against secondary care or hospital-based recruitment.

The UK Biobank offers the opportunity to learn lessons of general relevance for large-scale volunteer-based studies.^9^ While the UK Biobank population as a whole has been well characterized, the genetic and phenotypic features of those with serious mental illness have not. Here, we investigate the extent to which schizophrenia as diagnosed in UK Biobank resembles schizophrenia in large genetic studies, as represented by those included in the Psychiatric Genomics Consortium (PGC) or as diagnosed in clinically ascertained samples. We compared genetic correlations of the UK Biobank with the PGC and compared polygenic risk scores (PRS), rates of copy number variation (CNV), and phenotypic features of individuals with schizophrenia in the UK Biobank with 4 independent UK-based samples. These findings are of general relevance to studies from other human biobanks, mental health cohorts defined from electronic health records, and other alternative sources.

## Methods

### Participants

Participants were included from the UK Biobank^10^ (approximately 500 000 individuals) and 4 schizophrenia sample collections (approximately 14 000 individuals from CLOZUK,^11^ 767 from CardiffCOGS,^12^ 648 from Cardiff F-Series,^12^ and 381 from Cardiff Affected Sib-Pairs^12^) (Table 1). Genetic analyses included all samples, and phenotypic analyses included all samples apart from CLOZUK. This study followed the Strengthening the Reporting of Genetic Association Studies (STREGA) reporting guideline.

**Table 1.** Cohort Descriptions

Characteristic	UK Biobank participants with schizophrenia	UK Biobank unaffected controls	CLOZUK	CardiffCOGS	Cardiff F-Series	Cardiff Affected Sib-Pairs	PGC Schizophrenia
Total, No.	1438	499 475	14 666	767	648	381	67 390 Cases and 94 015 controls
Sex, %							
Female	38.2	54.4	28.8	29.6	30.0	31.2	34.9
Male	61.8	45.6	71.2	70.4	70.0	68.8	65.1
Age at recruitment (SD), y	54.7 (8.3)	56.5 (8.1)	37.7 (11.9)^a^	42.9 (12.3)	42.0 (12.0)	41.5 (12.6)	Unknown
Ancestry description	Multiancestry	Multiancestry	Multiancestry	European ancestry	European ancestry	European ancestry	Multiancestry
Treatment resistance	Unknown	NA	100% TRS	56.2% TRS	27.5% TRS	40.7% TRS	Estimated minimum of 19%
Ascertainment	Volunteer-based biobank	Volunteer-based biobank	Anonymously ascertained from routine clozapine monitoring services	Clinically ascertained	Clinically ascertained	Clinically ascertained affected sibling pairs	Primarily clinically ascertained

Abbreviations: NA, not applicable; PGC, Psychiatric Genomics Consortium; TRS, treatment-resistant schizophrenia.

^a^ CLOZUK age at recruitment is estimated from CLOZUK2 only and from the age at registration with Leyden Delta’s monitoring system.

UK Biobank is a biomedical database and research resource of approximately 500 000 individuals from across the UK aged 40 to 69 years at recruitment (between 2006 and 2010).^10^ There are 4 sources from which a schizophrenia diagnosis can be detected in UK Biobank: self-report (field identifiers 20002 and 20544), *International Statistical Classification of Diseases and Related Health Problems, Tenth Revision* (*ICD*-*10*) code F20 medical record diagnosis from hospital admissions (field identifiers 41202 and 41204) or death records (field identifiers 40001 and 40002), or an equivalent read code from primary care records (field identifier 130875). eAppendix 1 in Supplement 1 further describes these sources. We defined schizophrenia in UK Biobank as a schizophrenia diagnosis reported from at least 1 of these sources. A total of 1438 participants met 1 or more of these criteria at the time of analysis (eTable 1 in Supplement 1), which was based on data extracted in July 2021. Controls were defined as participants who had no indication of a psychotic disorder from the above sources (*ICD*-*10* codes F21-29 inclusive). The North-West Multi-Centre Ethics Committee granted ethical approval to UK Biobank, and all participants provided written informed consent. This study was conducted under UK Biobank project numbers 13310 and 14421.

CLOZUK is an anonymized sample of approximately 14 000 individuals taking clozapine in the UK with a diagnosis of treatment-resistant schizophrenia, as previously described.^11^ CardiffCOGS (n = 767), Cardiff F-Series (n = 648), and Cardiff Affected Sib-Pairs (n = 381) participants were recruited from community, inpatient, and voluntary mental health services in the UK.^12^ The Cardiff Affected Sib-Pairs sample includes families with 2 or more siblings diagnosed with schizophrenia (or schizoaffective disorder, provided one of the siblings had schizophrenia). *ICD*-*10* code F20 or *DSM*-*IV* schizophrenia diagnoses in CardiffCOGS, Cardiff F-Series, and Cardiff Affected Sib-Pairs were based on *Schedules for Clinical Assessment in Neuropsychiatry*^13^ interviews and lifetime psychiatric clinical case notes. All schizophrenia sample collections received UK National Research Ethics Service approval, and study participants provided written informed consent.

### Statistical Analysis

#### Comparison With PGC

We conducted a genome-wide association study (GWAS) of schizophrenia in the UK Biobank and used the results to calculate genetic correlations with the PGC samples. The GWAS compared participants in UK Biobank with schizophrenia with participants without any mental or behavioral disorder (defined as *ICD*-*10* codes F00-F99 in field category 1712) to circumvent artificial enrichments in the genetic correlations with other psychiatric conditions. UK Biobank participants were genotyped on either the UK Biobank Axiom or the UK BiLEVE Axiom purpose-built arrays. Standard quality-control procedures were applied prior to imputation using the Haplotype Reference Consortium panel, as previously described.^14,15^ Single-nucleotide variants (SNVs) were excluded using PLINK version 2.0^16^ in line with thresholds used by the PGC^17^: minor allele frequency less than 0.01, Hardy-Weinberg equilibrium *P* values less than 1.00 × 10^−6^ using the midp and keep-fewhet options for multipopulation datasets, imputation quality information score less than 0.9, and SNV call rate less than 0.95. Individuals with SNV missingness greater than 0.05 were excluded.

Association testing was based on the Scalable and Accurate Implementation of Generalized Mixed Model (SAIGE) method.^18^ The SAIGE method is appropriate when case-control numbers are unbalanced and/or in the context of population structure. The null logistic model was conducted on a reduced dataset of relatively independent SNVs (n = 90 684), created using PLINK’s^16^ pruning procedure (*r*^2^ less than .05 and 500-kilobase window). Covariates included in the null logistic model were the first 5 principal components, plus any principal components from the first 20 that were associated with schizophrenia, genotyping array, self-reported ethnicity, sex, and age at interview (individuals with schizophrenia were younger than unaffected controls). The leave-1-chromosome-out option was implemented to account for related individuals. Post-GWAS processing was conducted using FUMA GWAS version 1.5.0 (Department of Complex Trait Genetics at VU University Amsterdam)^19^ to annotate and visualize the results.

Genetic correlations were calculated using linkage disequilibrium score regression^20,21^ between the schizophrenia GWAS in UK Biobank and GWAS for schizophrenia,^17^ bipolar disorder,^22^ major depressive disorder (MDD),^23^ attention-deficit/hyperactivity disorder,^24^ autism spectrum disorder,^25^ anorexia nervosa,^26^ cannabis use disorder,^27^ alcohol use disorder,^28^ and intelligence.^29^ Corresponding genetic correlations were also calculated for the PGC GWAS for schizophrenia, and differences with UK Biobank schizophrenia results assessed via χ^2^ tests. Unless otherwise stated, significance was set at *P* < .05. All *P* values were 2-tailed.

A schizophrenia PRS was calculated in UK Biobank using a method consistent with the PGC^17^ to allow comparison of the variance explained in schizophrenia case-control with the PGC and UK Biobank. The PRS was calculated via a clumping and thresholding approach in PRSicev2^30^ for those of European genetic ancestry, as previously described.^14^

#### Comparison With Clinically Ascertained Cohorts

We compared PRSs, rates of CNVs, and phenotypic features between cohorts. Unless otherwise stated, statistical analyses were conducted in R version 4.2.1 (The R Foundation).

##### PRS

CardiffCOGS, Cardiff F-Series, and Cardiff Affected Sib-Pairs were genotyped on the Illumina HumanOmniExpress version 8 or 12 (Illumina). CLOZUK samples were genotyped on either the Illumina HumanOmniExpress-12 or Illumina HumanOmniExome-8 array.^11^ For Cardiff University samples, quality control and imputation using the Haplotype Reference Consortium panel was conducted as part of the DRAGON-Data protocol.^31^ The steps taken to combine the genetic data from UK Biobank and our clinically ascertained cohorts to calculate PRS are described in eAppendix 2 in Supplement 1. A subset of SNVs from this combined dataset with low levels of linkage disequilibrium (*r*^2^ less than 0.2 at 500-kilobase window) were used to identify unrelated individuals and to calculate principal components. The randomly selected unrelated individuals were identified using the Kinship-Based Inference for GWAS (KING) robust kinship estimator in PLINK. A kinship cutoff of 0.044 was used, equivalent to removing third-degree relatives. Principal components were calculated using PC-AiR^32^ from the GENESIS package. Plots comparing principal components by study showed no evidence of differences by study and genotyping array (eFigure 1 in Supplement 1).

PRS were calculated for schizophrenia,^17^ bipolar disorder,^22^ MDD,^33^ and intelligence^29^ based on GWAS summary statistics that did not overlap with those in the present study. In collaboration with Cardiff University after permission from UK Biobank under project number 13310, the Schizophrenia Working Group of the PGC generated a custom GWAS that excluded UK Biobank participants (based on checksums derived from the genomic data) and the Cardiff University samples. Intelligence summary statistics were derived as part of a related project.^14,29^ Bipolar disorder^22^ and MDD^33^ summary statistics were obtained from the PGC. Summary statistics were cleaned using summaRygwasqc.^34^ Using all SNVs in the combined dataset, we used PRS-CS^35^ and PLINK to calculate the PRS using the EUR UK Biobank reference dataset, 10 000 burn-in iterations, 25 000 Markov chain Monte Carlo iterations, and a φ value of 1 for schizophrenia and the default φ value for intelligence, bipolar disorder, and MDD.

We scaled the PRS in all samples using principal components^36^ to allow comparisons regardless of ancestry. This approach was effective as demonstrated by eFigure 2 in Supplement 1, which displays the adjusted and unadjusted PRS in biogeographical genetic ancestry groups^37^ (eAppendix 3 and eFigure 3 in Supplement 1). Pairwise comparisons for the PRS were made between individuals with schizophrenia in UK Biobank and other samples using logistic regression controlling for sex. A Bonferroni correction was applied (20 tests; *P* < .0025) to determine significance. We repeated analyses in individuals of European genetic ancestry as defined by biogeographical grouping to ensure results were consistent.

##### Schizophrenia-Associated CNVs

Details of CNV calling have been described for CLOZUK,^38,39^ UK Biobank,^40^ and CardiffCOGS.^39^ The Cardiff F-Series and Cardiff Affected Sib-Pairs samples were called as part of the DRAGON-Data protocol.^31^ One member from each third-degree (or more closely) related pair within each dataset was removed at random. As the CNVs of interest are rare, we combined the participants from CardiffCOGS, Cardiff F-Series, and Cardiff Sib-pairs. Analyses were restricted to individuals of European genetic ancestry, as defined in eAppendix 4 in Supplement 1, due to the low numbers of observations and because most individuals in the clinically ascertained schizophrenia samples were of European genetic ancestry. We compared the number of individuals in UK Biobank with schizophrenia that carried any of 12 schizophrenia-associated CNVs^38^ (eTable 2 in Supplement 1) to the other samples using pairwise Firth logistic regressions covarying for sex.

##### Schizophrenia-Related Phenotypes

UK Biobank participants with schizophrenia were compared with those in CardiffCOGS, Cardiff F-Series, and Cardiff Affected Sib-Pairs and with controls in UK Biobank for phenotypes known to be related to schizophrenia, including demographic characteristics, education attainment, cognitive ability, and known psychiatric and physical comorbidities of schizophrenia. CLOZUK was not included due to the absence of relevant phenotypic data. It was not possible to include phenotypes from UK Biobank’s mental health questionnaire due to the low completion rate in individuals with schizophrenia (14.5%), a return rate much lower than for the UK Biobank as a whole (31.5%). Comparisons were made only when equivalent definitions were available across samples, ie, were assessed using similar wording on their respective questionnaires and/or where responses could be harmonized into comparable categories. eTable 3 in Supplement 1 details each phenotype and its definition in each sample. Pairwise comparisons were calculated between schizophrenia cases in UK Biobank and the other samples using logistic regression controlling for sex and age at recruitment. A Bonferroni correction for the number of tests was applied (52 tests; *P* < 9.62 × 10^−4^). Year of birth was also included for the education variables. Secondary analyses were conducted restricted to those of European genetic ancestry (eMethods 3 in Supplement 1).

## Results

The sample of 517 375 participants included 1438 UK Biobank participants with schizophrenia (550 [38.2%] female; mean [SD] age, 54.7 [8.3] years), 499 475 UK Biobank controls (271 884 [54.4%] female; mean [SD] age, 56.5 [8.1] years), and 4 schizophrenia research samples (4758 [28.9%] female; mean [SD] age, 38.2 [21.0] years). The 1438 individuals (0.3% of UK Biobank total sample) in UK Biobank were identified with a schizophrenia diagnosis from at least 1 of the available sources: 1102 (76.7%) from hospital records, 708 (49.2%) from self-report, 75 (5.2%) from primary care records, and 23 (1.6%) from death records. A total of 462 individuals had more than 1 source of diagnosis (eTable 1 in Supplement 1).

### Comparison With PGC

**Figure 1.**
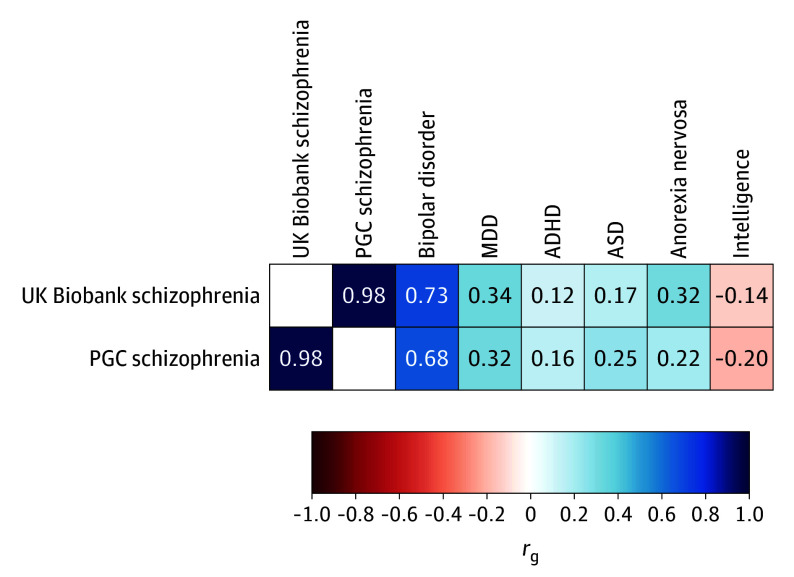
Genetic Correlations Genetic correlations between the schizophrenia genome-wide association study in UK Biobank and Psychiatric Genomics Consortium (PGC) schizophrenia,^17^ bipolar disorder,^22^ major depressive disorder (MDD),^23^ attention-deficit/hyperactivity disorder (ADHD),^24^ autism spectrum disorder (ASD),^25^ anorexia nervosa,^26^ and intelligence.^29^ Comparison correlations with PGC schizophrenia are given in second row. The color of each box indicates the magnitude of the correlation. Statistics and statistical comparison between the correlations are provided in eTable 5 in Supplement 1.

After quality control, a GWAS including 1363 individuals with schizophrenia and 358 774 controls from UK Biobank did not identify any genome-wide significantly associated loci (threshold *P* < 5 × 10^−8^), as expected for a case sample of this size (genomic control λ = 1.03; eFigures 4 and 5 in Supplement 1). Schizophrenia in the UK Biobank had a genetic correlation with the latest PGC schizophrenia GWAS^17^ that was close to 1 (*r*_g_ = 0.98; SE, 0.18). The genetic correlations between UK Biobank schizophrenia and bipolar disorder (*r*_g_ = 0.73; SE, 0.14), MDD (*r*_g_ = 0.34; SE, 0.08), intelligence (*r*_g_ = −0.14; SE, 0.06), or between any of the other neuropsychiatric disorders were not significantly different from the genetic correlations between those traits and the latest PGC schizophrenia GWAS study (Figure 1; eTable 4 in Supplement 1).

The schizophrenia PRS calculated from the PGC GWAS was associated with schizophrenia case-control status within those of European genetic ancestry in UK Biobank (liability *R*^2^ = 6.8%; odds ratio [OR], 2.04; 95% CI, 1.92-2.17; *P* = 6.05 × 10^−110^). A liability *R*^2^ of 6.8% would be the 54th highest value out of 76 comparable samples in the latest PGC GWAS.^17^

### Comparisons With Clinically Ascertained Cohorts

#### PRS

**Figure 2.**
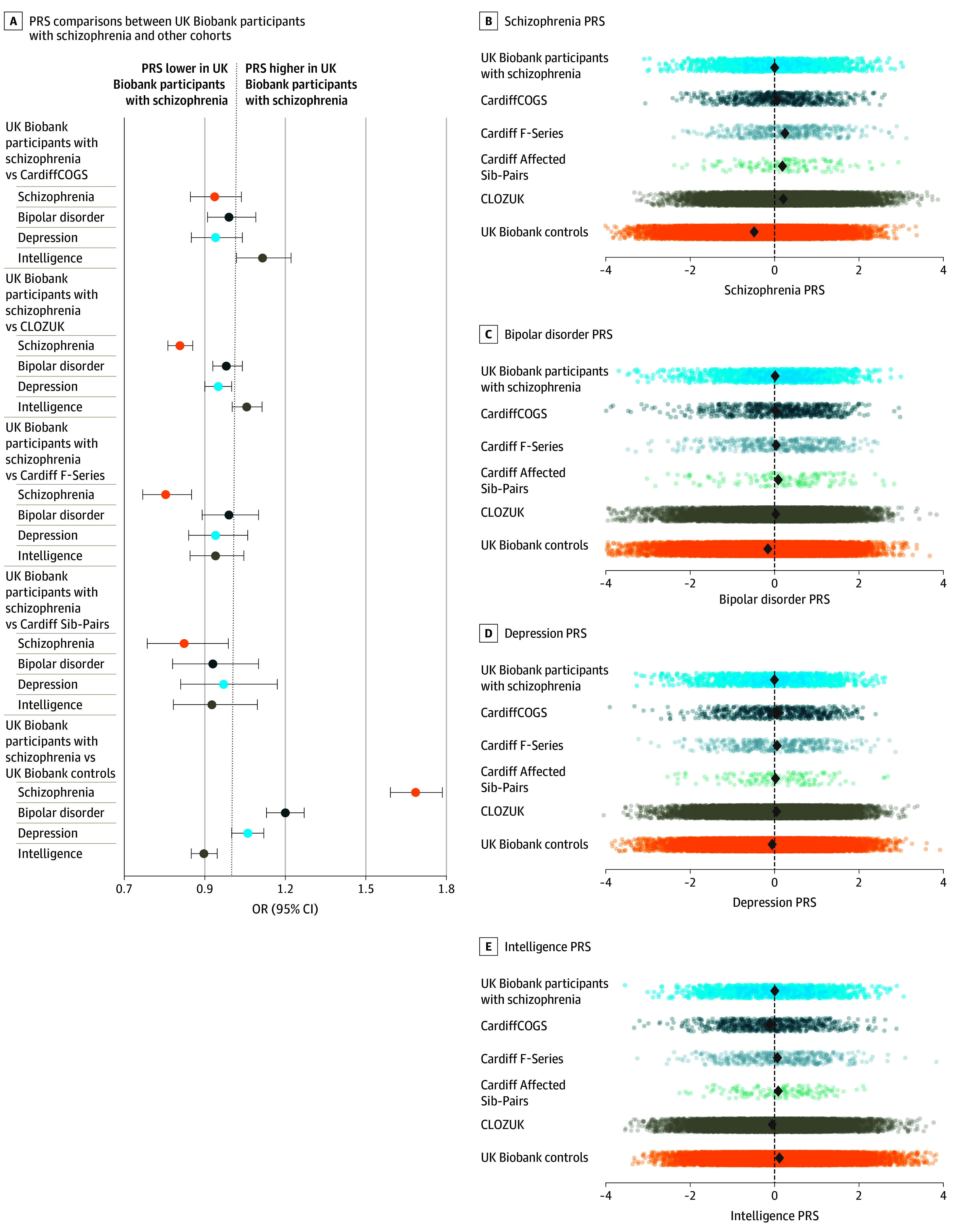
Polygenic Risk Comparisons Between Cohorts A, Odds ratios (ORs) from the polygenic risk score (PRS) comparisons between UK Biobank participants with schizophrenia and the other cohorts (eTable 5 in Supplement 1). The vertical dotted line corresponds to 1 (null association). Error bars indicate 95% CIs. B-E, Distribution of each PRS for each cohort. The vertical dotted line represents the mean PRS in UK Biobank participants with schizophrenia. Diamonds represent the mean PRS for each sample.

Compared with the clinically ascertained cohort, participants with schizophrenia in UK Biobank had on average a lower schizophrenia PRS, significantly so compared with CLOZUK, Cardiff F-Series, and Cardiff Affected Sib-Pairs (Figure 2; eTable 5 in Supplement 1). The intelligence PRS for individuals with schizophrenia in UK Biobank was higher compared with CLOZUK and CardiffCOGS but not compared with Cardiff F-Series or Cardiff Affected Sib-Pairs (Figure 2; eTable 5 in Supplement 1). These results were consistent when restricting analyses to individuals of European genetic ancestry (eTable 6 in Supplement 1). Schizophrenia case-control status in UK Biobank individuals of all genetic ancestries was associated with the schizophrenia PRS (OR, 1.69; 95% CI, 1.59-1.78; *P* = 3.79 × 10^−71^), bipolar disorder PRS (OR, 1.20; 95% CI, 1.13-1.27; *P* = 4.29 × 10^−10^), and intelligence PRS (OR, 0.89; 95% CI, 0.85-0.94; *P* = 3.34 × 10^−5^) but not the MDD PRS (OR, 1.06; 95% CI, 1.00-1.12; *P* = .045) (Figure 2; eTable 5 in Supplement 1).

#### CNV

A total of 16 of 964 UK Biobank individuals with schizophrenia (1.6%) had a schizophrenia-associated CNV compared with 3153 of 388 371 controls (0.8%; OR, 2.07; 95% CI, 1.22-3.25; *P* = .009). eTable 2 in Supplement 1 lists the number of carriers per CNV and cohort. The CNV rate for UK Biobank participants with schizophrenia was lower than for the CLOZUK participants with schizophrenia (1.6% vs 324 of 11 850 [2.7%]; OR, 0.60; 95% CI, 0.35-0.95; *P* = .03). A similar pattern was observed for the comparison between the UK Biobank and the combined sample of CardiffCOGS, Cardiff F-Series, and Cardiff Affected Sib-Pairs (1.6% vs 26 of 1074 [2.4%]; OR, 0.63; 95% CI, 0.33-1.17; *P* = .14); although not significant, the sample size in that analysis means power to demonstrate a true difference is low.

#### Schizophrenia-Related Phenotypes

**Figure 3.**
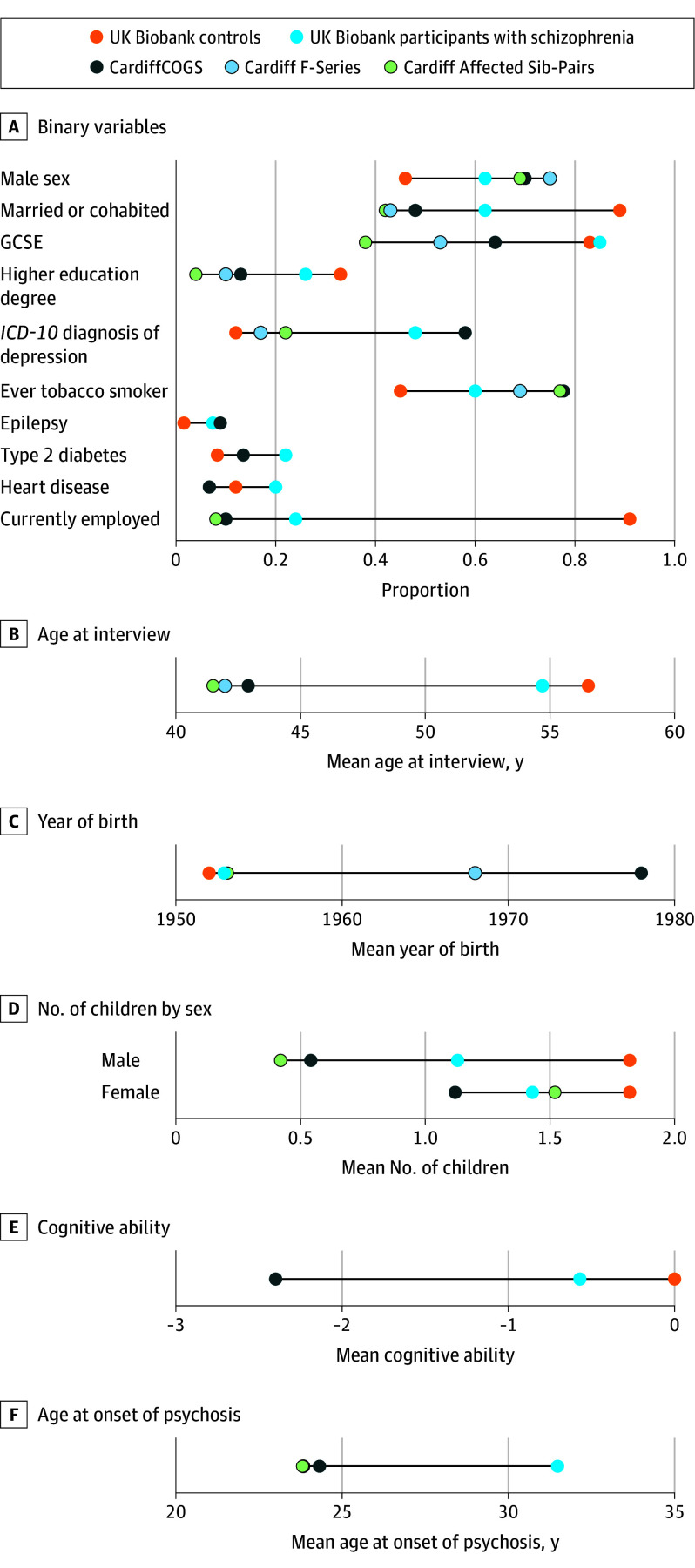
Phenotypic Comparisons Between Cohorts Cleveland plot of proportion (binary variables) or mean (continuous variables) values for each phenotype for each study. GCSE indicates General Certificate of Secondary Education; *ICD*-*10*, *International Statistical Classification of Diseases and Related Health Problems, Tenth Revision*.

Phenotypic features of the samples are displayed in Figure 3 and eTable 7 in Supplement 1. Rates of comorbid affective diagnoses for the UK Biobank participants with schizophrenia are described in eAppendix 5 in Supplement 1. Compared with the clinically ascertained schizophrenia samples, UK Biobank participants had patterns consistent with lower severity of illness (Table 2); they were less likely to be male, and male participants were more likely to have children (there was no difference in female participants). All cognitive indices, including educational attainment and cognitive ability, were higher in UK Biobank participants with schizophrenia. These participants had higher rates of current employment and an older self-reported age of onset of psychosis compared with the clinically ascertained samples. UK Biobank participants with schizophrenia had lower rates of smoking but equivalent rates of comorbid physical illness once age was adjusted for.

**Table 2.** Phenotypic Comparisons Between UK Biobank Participants With Schizophrenia and Each Cohort^a^

Phenotype	UK Biobank participants with schizophrenia vs UK Biobank controls		UK Biobank participants with schizophrenia vs CardiffCOGS		UK Biobank participants with schizophrenia vs Cardiff F-Series		UK Biobank participants with schizophrenia vs Cardiff Affected Sib-Pairs	
	OR (95% CI)	*P* value	OR (95% CI)	*P* value	OR (95% CI)	*P* value	OR (95% CI)	*P* value
Male sex	1.95 (1.75-2.17)	1.30 × 10^−34^	0.81 (0.66-1.01)	.06	0.86 (0.69-1.09)	.22	0.91 (0.66-1.26)	.58
Age at interview (per 1-y older)	0.80 (0.76-0.84)	1.22 × 10^−18^	2.51 (2.30-2.75)	2.29 × 10^−89^	2.38 (2.17-2.60)	2.21 × 10^−81^	3.31 (2.85-3.85)	2.53 × 10^−54^
Year of birth (per 1-y older)	1.26 (1.20-1.33)	2.17 × 10^−19^	0.33 (0.30-0-36)	2.02 × 10^−104^	0.80 (0.74-0.86)	4.44 × 10^−9^	1.01 (0.91-1.13)	.78
Married or cohabited	0.19 (0.16-0.23)	1.41 × 10^−78^	0.89 (0.68-1.16)	.39	1.19 (0.91-1.56)	.21	1.24 (0.88-1.77)	.22
Male children (per 1 child more)	0.64 (0.61-0.69)	4.39 × 10^−43^	1.18 (1.04-1.33)	.009	NA	NA	1.30 (1.08-1.56)	.006
Female children (per 1 child more)	0.75 (0.70-0.81)	7.44 × 10^−14^	0.96 (0.83-1.11)	.60	NA	NA	0.88 (0.75-1.05)	.16
Currently employed	0.03 (0.03-0.03)	<1 × 10^−216^^b^	3.33 (2.38-4.64)	1.78 × 10^−12^	NA	NA	2.98 (1.71-5.17)	1.11 × 10^−4^
GCSE	1.20 (1.03-1.38)	.02	4.01 (3.09-5.21)	2.59 × 10^−25^	5.30 (4.23-6.65)	1.76 × 10^−47^	8.78 (6.56-11.76)	2.52 × 10^−48^
Higher-education degree	0.66 (0.59-0.75)	2.61 × 10^−11^	2.48 (1.85-3.32)	1.23 × 10^−9^	3.23 (2.39-4.36)	2.53 × 10^−14^	9.74 (4.95-19.16)	4.24 × 10^−11^
Cognitive ability^c^	0.54 (0.49-0.60)	6.17 × 10^−35^	5.74 (4.59-7.18)	8.85 × 10^−53^	NA	NA	NA	NA
Ever tobacco smoker	1.78 (1.60-1.99)	7.36 × 10^−26^	0.48 (0.38-0.60)	3.49 × 10^−10^	0.75 (0.59-0.95)	.02	0.48 (0.32-0.74)	7.21 × 10^−4^
*ICD*-*10* diagnosis of depression^d^	7.57 (6.82-8.41)	<1 × 10^−216^^b^	0.68 (0.55-0.84)	3.27 × 10^−4^	4.63 (3.55-6.03)	1.01 × 10^−29^	3.35 (2.37-4.74)	8.63 × 10^−12^
Epilepsy	4.77 (3.91-5.81)	1.08 × 10^−53^	0.93 (0.63-1.37)	.73	NA	NA	NA	NA
Type 2 diabetes	3.31 (2.92-3.77)	1.93 × 10^−75^	1.30 (0.89-1.73)	.07	NA	NA	NA	NA
Heart disease	1.93 (1.68-2.21)	3.71 × 10^−21^	2.49 (1.65-3.75)	1.23 × 10^−5^	NA	NA	NA	NA
Onset of psychosis	NA	NA	1.53 (1.34-1.74)	3.83 × 10^−10^	1.74 (1.50-2.01)	3.86 × 10^−13^	1.92 (1.55-2.37)	2.52 × 10^−9^

Abbreviations: GCSE, General Certificate of Secondary Education; *ICD*-*10*, *International Statistical Classification of Diseases and Related Health Problems, Tenth Revision*; NA, not available; OR, odds ratio.

^a^ Results from pairwise regressions between UK Biobank participants with schizophrenia and each of the other samples for each phenotype assessed. ORs refer to risk in UK Biobank participants with schizophrenia; if greater than 1, this indicates higher rates (or higher values for continuous phenotypes) of said phenotype in these participants compared with the other sample. Corresponding proportions and means are presented in eTable 7 in Supplement 1. Onset of psychosis (n = 638) and cognitive ability (n = 451) were only available for a subset of UK Biobank participants with schizophrenia.

^b^ Below the threshold the software could calculate.

^c^ Cognitive ability defined in the UK Biobank from fluid intelligence (field identifier 20016) and from MATRICS composite score in CardiffCOGS (eTable 3 in Supplement 1).

^d^ Depression defined as *ICD*-*10* codes F32 and F33.

Compared with controls, UK Biobank participants with schizophrenia were more likely to be male, less likely to have been married or to have had children, had lower educational outcomes indexed by a high school qualification (General Certificate of Secondary Education) or a higher-education degree, and had lower cognitive ability as measured by fluid intelligence (Figure 3; Table 2). They had higher rates of depression, tobacco use, epilepsy, heart disease, and type 2 diabetes. Individuals with schizophrenia of working age had lower levels of current employment.

There was no evidence of an underrepresentation of individuals with schizophrenia from ethnic minority groups compared with CLOZUK or controls (eTables 8 and 9 in Supplement 1). All phenotypic analyses were consistent in analyses restricted to those of European genetic ancestry.

## Discussion

We compared individuals with schizophrenia from UK Biobank with those with schizophrenia in the PGC and with 4 clinically ascertained schizophrenia research samples. Participants with schizophrenia in UK Biobank had the genomic and phenotypic features expected from previous research but consistent with them being less severely affected.

Schizophrenia in UK Biobank had a genetic correlation of 0.98 (SE, 0.18) with the latest PGC schizophrenia GWAS.^17^ Schizophrenia PRS explained 6.8% of the variance in liability for schizophrenia case-control status in those of European genetic ancestry in UK Biobank, which, while lower than the variance explained across the PGC samples as a whole (7.3% in all samples; 8.1% in those of European genetic ancestry),^17^ is within the range of other schizophrenia PGC samples. The association between schizophrenia PRS and schizophrenia case-control status in UK Biobank (all samples: OR, 1.69; 95% CI, 1.59-1.78; European genetic ancestry: OR, 2.04; 95% CI, 1.92-2.17) was also comparable with estimates from the PsycheMERGE consortium^41^ (OR, 1.55; 95% CI, 1.39-1.72) and US Veterans Affairs Health Care System^42^ (OR, 1.56; 95% CI, 1.52-1.61). A 2023 study found the average schizophrenia PRS did not differ between individuals with schizophrenia identified via different diagnostic sources in UK Biobank.^43^ In addition, we observed phenotypic associations expected of schizophrenia, such as an excess of male sex, lower cognitive outcomes, low rates of current employment, and rates of physical health comorbidities in UK Biobank participants with schizophrenia comparable with epidemiological samples of schizophrenia.^44^

After PGC schizophrenia, the next highest genetic correlation for UK Biobank participants with schizophrenia was with bipolar disorder^22^ (0.73; SE, 0.14), the psychiatric disorder most genetically correlated with schizophrenia, and correlations with other psychiatric disorders were consistent with those from the PGC schizophrenia GWAS,^17^ indicating that the genetics of the schizophrenia diagnosis in UK Biobank is compatible with others typically used in genomic studies. This is further supported by the strength of the schizophrenia PRS (OR, 1.69; 95% CI, 1.59-1.78) association with schizophrenia case-control status in contrast to bipolar disorder (OR, 1.20; 95% CI, 1.13-1.27) or MDD (OR, 1.06; 95% CI, 1.00-1.12).

Comparisons with clinically ascertained schizophrenia cohorts indicated that those with schizophrenia in UK Biobank likely represent less severely affected cases. Compared with the other schizophrenia samples, UK Biobank participants with schizophrenia had lower rates of male sex, higher cognitive ability and educational attainment, lower rates of smoking, older age of onset of psychosis, and higher current employment. Further, the rate of schizophrenia-associated CNVs and the schizophrenia PRS was lower in UK Biobank cases compared with Cardiff schizophrenia samples, although the latter is well within the range of values for individual studies included in the PGC.^17^

These findings reported here almost certainly reflect, in part, ascertainment differences. It is likely that focusing on clinically ascertained samples in research may bias estimates toward more severe outcomes and that UK Biobank could offer an opportunity to study those with better outcomes. In addition, we found ethnic minority groups to be equally represented in UK Biobank participants with schizophrenia compared with CLOZUK participants or controls. While biobanks have advantages, they also have biases and tend to undersample individuals with serious mental illness and hence are an inefficient way to recruit large numbers of representative schizophrenia cases. Further, many phenotypes routinely collected in clinical schizophrenia cohorts were not available in UK Biobank, and most people with schizophrenia did not complete online follow-up questionnaires, such as the mental health questionnaire. Given future studies of the genetic basis of heterogeneity in schizophrenia will require both large numbers and high-quality assessments, targeted cohorts will still be needed, but these could be enhanced by the use of linked electronic medical records.^45^ There is an inevitable added cost for studies recruiting individuals with serious forms of mental illness, but this is essential if we are to base our research on representative samples and be able to generalize our findings.

Our findings have important implications for schizophrenia research conducted within and outside of UK Biobank. They indicate the need to integrate both cases recruited from secondary mental health services, which will be weighted toward more severe outcomes, and those from biobank resources, which will capture a higher proportion of less severely affected cases, to encapsulate the full spectrum of schizophrenia.

### Limitations

This study has limitations. In this article, we selected a pragmatic definition of schizophrenia that will be applicable to other biobank studies. More sophisticated definitions based on diagnostic algorithms or machine learning approaches are being developed and could offer further advantages to the field in the future. This study was conducted within the UK, and many of the individuals were identified from linked medical records, so results will need replication to ensure generalizability to other biobanks, countries, and health care settings. The small sample size for CNV analyses meant that power to demonstrate significant differences was low.

## Conclusions

Individuals with schizophrenia in UK Biobank have genomic and phenotypic features consistent with expectations for those with a diagnosis of schizophrenia but represent those less severely affected. The inclusion of such cases in wider schizophrenia studies has the potential to enhance representation of the full spectrum of illness severity.

## Notes

Supplement 1. **eAppendix 1.** Sources of Schizophrenia Diagnosis in UK Biobank **eAppendix 2.** Genetic Data Quality Control for Combined Dataset **eAppendix 3.** Biogeographical Genetic Ancestry Groups **eAppendix 4.** Definition of European Genetic Ancestry in CNV Analyses **eAppendix 5.** Comorbid Affective Diagnoses in UK Biobank **eFigure 1.** Assessment of Batch Effects **eFigure 2.** PRS Ancestry Adjustment **eFigure 3.** Biogeographical Genetic Ancestries Groupings **eFigure 4.** Manhattan Plot for Schizophrenia in UK Biobank GWAS **eFigure 5.** QQ Plot for Schizophrenia in UK Biobank GWAS **eTable 1.** Source of Schizophrenia Diagnosis in UK Biobank **eTable 2.** Number of CNVs per Cohort **eTable 3.** Phenotype Descriptions **eTable 4.** Genetic Correlations With Schizophrenia in UK Biobank **eTable 5.** Polygenic Risk Score Comparisons Between Cohorts **eTable 6.** PRS Analyses in Individuals of European Genetic Ancestry **eTable 7.** Phenotypic Characteristics per Cohort **eTable 8.** Genetic Ancestry Differences Between Case Cohorts **eTable 9.** Self-Reported Ethnicity and Schizophrenia in UK Biobank
eReferences.

Supplement 2. Data Sharing Statement
