# Supplementary material for: Genetic and Phenotypic Features of Schizophrenia in the UK Biobank
Source: JAMA Psychiatry. 2024 Mar 27;81(7):681–90. doi: 10.1001/jamapsychiatry.2024.0200 (PMC10974692; doi:10.1001/jamapsychiatry.2024.0200)
Supplement: Supplement 1. — eAppendix 1. Sources of Schizophrenia Diagnosis in UK Biobank eAppendix 2. Genetic Data Quality Control for Combined Dataset eAppendix 3. Biogeographical Genetic Ancestry Groups eAppendix 4. Definition of European Genetic Ancestry in CNV Analyses eAppendix 5. Comorbid Affective Diagnoses in UK Biobank eFigure 1. Assessment of Batch Effects eFigure 2. PRS Ancestry Adjustment eFigure 3. Biogeographical Genetic Ancestries Groupings eFigure 4. Manhattan Plot for Schizophrenia in UK Biobank GWAS eFigure 5. QQ Plot for Schizophrenia in UK Biobank GWAS eTable 1. Source of Schizophrenia Diagnosis in UK Biobank eTable 2. Number of CNVs per Cohort eTable 3. Phenotype Descriptions eTable 4. Genetic Correlations With Schizophrenia in UK Biobank eTable 5. Polygenic Risk Score Comparisons Between Cohorts eTable 6. PRS Analyses in Individuals of European Genetic Ancestry eTable 7. Phenotypic Characteristics per Cohort eTable 8. Genetic Ancestry Differences Between Case Cohorts eTable 9. Self-Reported Ethnicity and Schizophrenia in UK Biobank eReferences. [file jamapsychiatry-e240200-s001.pdf]

Supplemental Online Content

Legge SE, Pardiñas AF, Woolway G, et al. Genetic and phenotypic features of schizophrenia in the UK Biobank. *JAMA Psychiatry*. Published online March 27, 2024. doi:10.1001/jamapsychiatry.2024.0200

eAppendix 1. Sources of Schizophrenia Diagnosis in UK Biobank

eAppendix 2. Genetic Data Quality Control for Combined Dataset

eAppendix 3. Biogeographical Genetic Ancestry Groups

eAppendix 4. Definition of European Genetic Ancestry in CNV Analyses

eAppendix 5. Comorbid Affective Diagnoses in UK Biobank

eFigure 1. Assessment of Batch Effects

eFigure 2. PRS Ancestry Adjustment

eFigure 3. Biogeographical Genetic Ancestries Groupings

eFigure 4. Manhattan Plot for Schizophrenia in UK Biobank GWAS

eFigure 5. QQ Plot for Schizophrenia in UK Biobank GWAS

eTable 1. Source of Schizophrenia Diagnosis in UK Biobank

eTable 2. Number of CNVs per Cohort

eTable 3. Phenotype Descriptions

eTable 4. Genetic Correlations With Schizophrenia in UK Biobank

eTable 5. Polygenic Risk Score Comparisons Between Cohorts

eTable 6. PRS Analyses in Individuals of European Genetic Ancestry

eTable 7. Phenotypic Characteristics per Cohort

eTable 8. Genetic Ancestry Differences Between Case Cohorts

eTable 9. Self-Reported Ethnicity and Schizophrenia in UK Biobank

eReferences.

This supplementary material has been provided by the authors to give readers additional information about their work.

© 2024 Legge SE et al. *JAMA Psychiatry*.

eAppendix 1. Sources of Schizophrenia Diagnosis in UK Biobank

We defined schizophrenia in UK Biobank as a schizophrenia diagnosis reported from at least one of; self-report (field IDs 20002/20544), ICD-10 F20 medical record diagnosis from hospital admissions (field IDs 41270/41202/41204) and death records (field IDs 40001/40002), or an equivalent read code from primary care records (field ID 130875). Full descriptions of these sources are available on the UK Biobank showcase (https://biobank.ndph.ox.ac.uk/ukb/).

Self-report: There were two occasions that participants were asked about a diagnosis of schizophrenia, the initial recruitment interview and during a subsequent online mental health questionnaire (MHQ). As part of the initial interview all participants were asked "have you been told by a doctor that you have any other serious illness or disabilities". Participants were not specifically prompted for a schizophrenia diagnosis, but if it was volunteered by the participant then this was recorded. Initiations via email in 2016/2017 were sent to 333,420 participants and the MHQ was completed by a total of 157,328 participants. As part of the questionnaire participants were asked "Have you ever been diagnosed with one or more of the following mental health problems by a professional, even if you don't have it currently?" and a list of mental health conditions presented including schizophrenia and psychotic disorders. 157 individuals who completed the MHQ self-reported a schizophrenia diagnosis. Further information can be found in field IDs 20002 and 20544.

Hospital admissions: Hospital records are provided separately for England, Wales and Scotland and come from National databases. These records date back to 1997 for England, 1998 for Wales and 1981 for Scotland. Not every participant will have hospital inpatient record, as not all have been admitted to hospital over this period covered but

© 2024 Legge SE et al. *JAMA Psychiatry*.

if participants access NHS hospital care, these records will be provided. In England the dataset is called Hospital Episode Statistics (HES) Admitted Patient Care (APC), which is provided by the Data Access Request Service (DARS) and managed by NHS England. In Wales, hospital admissions data is provided by the Secure Anonymised Information Linkage (SAIL) databank at the University of Swansea, managed by NHS Wales Informatics Service's Information Services Division (ISD). This dataset is called the Patient Episode Database for Wales (PEDW) Admitted Patient Care (APC). Data on hospital admissions for Scotland are provided by Public Health Scotland. There are two datasets linked; the General Acute Inpatient and Day Case – Scottish Morbidity Record (SMR01), and the Mental Health Inpatient and Day Case – Scottish Morbidity Record (SMR04). Further information about hospital admissions can be found in UK Biobank resource 138438.

Death records: Data on UK Biobank participants who have died is provided by NHS England for participants in England and Wales and from the NHS central register (NHSCR), part of the National Records of Scotland, for participants in Scotland. This data is sent to UK Biobank on a quarterly basis. Further information can be found in UK Biobank resource 115559.

Primary care: Primary care data was available for approximately 45% of UK Biobank participants at the time of data analysis. There is no national system in the UK for collecting or sharing primary care data. Further information can be found in UK Biobank resource 591.

© 2024 Legge SE et al. *JAMA Psychiatry*.

eAppendix 2. Genetic Data Quality Control for Combined Dataset

A subset of unaffected participants from UK Biobank was selected for computational efficiency (*n*=~20,000), prioritising inclusion of a broad range of genetic ancestries for the purposes of the post-hoc ancestry adjustment and to match to cases. The 1000 Genomes Project Phase 3 sample (*n*=2504) was also included for the purposes of the post-hoc ancestry adjustment (see below). All datasets were converted to PLINK best guess genotype files. The same quality control exclusions were applied as in the GWAS analyses; minor allele frequency (MAF) < 0.01, Hardy-Weinberg equilibrium (HWE) p-value < $1x10^{-6}$ using the 'midp' and 'keep-fewhet' options for multi-population datasets, imputation INFO score < 0.9, SNP call rate < 0.95. Individuals with SNP missingness > 0.05 were excluded. Given that SNP overlap between the imputed datasets was adequate (*n*=3,191,491), the datasets were merged, restricting to overlapping SNPs. The same quality control thresholds were then re-applied to the combined dataset leaving a total of 46,579 individuals.

© 2024 Legge SE et al. *JAMA Psychiatry*.

eAppendix 3. Biogeographical Genetic Ancestry Groups

Using the merged dataset for polygenic risk score analyses, global ancestry inference for all samples was performed using the linear discriminant analysis (LDA) method, as previously described[1], with the following modifications. First, to increase the robustness of the overall procedure, we replaced the original Human Genome Diversity Project (HGDP) training reference panel (*n*=930[2,3]) with a larger and more diverse sample from the Allen Ancient DNA Resource (AADR) (https://reich.hms.harvard.edu/allen-ancient-dna-resource-aadr-downloadable-genotypes-present-day-and-ancient-dna-data; v50). This AADR sample was restricted to contemporaneous individuals and any sample duplicates were removed (*n*=6096).

Second, for consistency with recently published literature on ancestry reporting for genomic research, we mapped each training AADR sample to the reference groups defined by Huddart et al[4]. While several of these groups match our previous work, they extend it to seven biogeographical groups (American, East Asian, European, Central/South Asian, Near Eastern, Oceanian, Sub-Saharan African), and two admixed groups (African American/Afro-Caribbean and Latino). For a graphical representation of how each group maps to the world's regions and countries please see Figure 1 in Huddart et al[4].

Third, due to the large number of samples included in this analysis, the PCA-based dimensionality reduction procedure for AIMs was based on the "randomised" algorithm implemented in the *SNPRelate* package[5] and used by the PC-Air procedure. This mirrors the "fastPCA" method[6].

Fourth, the best probability threshold for determining an ancestral class was not defined as a fixed value, but determined within each ancestry by assessing the probability of a

correct vs. incorrect inference in the training AADR dataset. This procedure used Youden's index as optimality criterion, as implemented in the "probably" R package (https://probably.tidymodels.org/).

Other than this, all other aspects of the ancestry inference pipeline, such as the AIM definition procedure, were performed following the original publication. In summary, the global LDA model was based on 13,662 AIMs collapsed to 39 PCs. Balanced accuracies (sens+spec/2[7]) for ancestry classification of these LDA models ranged between 85.65% and 99.95% as estimated by 10-fold repeated cross-validation.

© 2024 Legge SE et al. *JAMA Psychiatry*.

eAppendix 4. Definition of European Genetic Ancestry in CNV Analyses

Biogeographical groupings (as described in Supplementary Methods 2) were predicted for all schizophrenia samples (CLOZUK, CardiffCOGS, Cardiff F-Series, Cardiff Affected Sib-Pairs) and for schizophrenia cases in UK Biobank and ~20,000 UK Biobank unaffected controls. European genetic ancestry was defined in the remaining UK Biobank control samples using the principal components as described in a previous publication[8]. A total of 22874 individuals in UK Biobank were defined using both approaches. Of the 8376 that were identified as having European genetic ancestry from the principal components method, 100% were classified as European by the biogeographical groupings. There were 2282 additional individuals that the biogeographical approach identified as European that the principal component method did not, indicating this approach is conservative.

© 2024 Legge SE et al. *JAMA Psychiatry*.

eAppendix 5. Comorbid Affective Diagnoses in UK Biobank

An ICD-10 major depressive disorder diagnosis was present in 695 (48.3%) of the individuals with schizophrenia in UK Biobank. Depression is a well-known co-morbidity of schizophrenia and was observed in CardiffCOGS (58%; Figure 3, eTable 7). A bipolar disorder or a manic episode was present for 248 (17.2%) individuals with schizophrenia. Of these, 62 (4.3% of total) had more ICD-10 hospital bipolar disorder codes than ICD-10 hospital schizophrenia codes and a further 73 (5.1% of total) had equal numbers of admissions for schizophrenia and bipolar disorder (1 each). This high level of co-morbidity has also been shown in other health records[9]. Of those that self-reported a schizophrenia diagnosis (n=708), 448 (63.3%) also had a hospital admission for schizophrenia and 67 (9.5%) an admission for bipolar disorder.

© 2024 Legge SE et al. *JAMA Psychiatry*.

eFigure 1. Assessment of Batch Effects

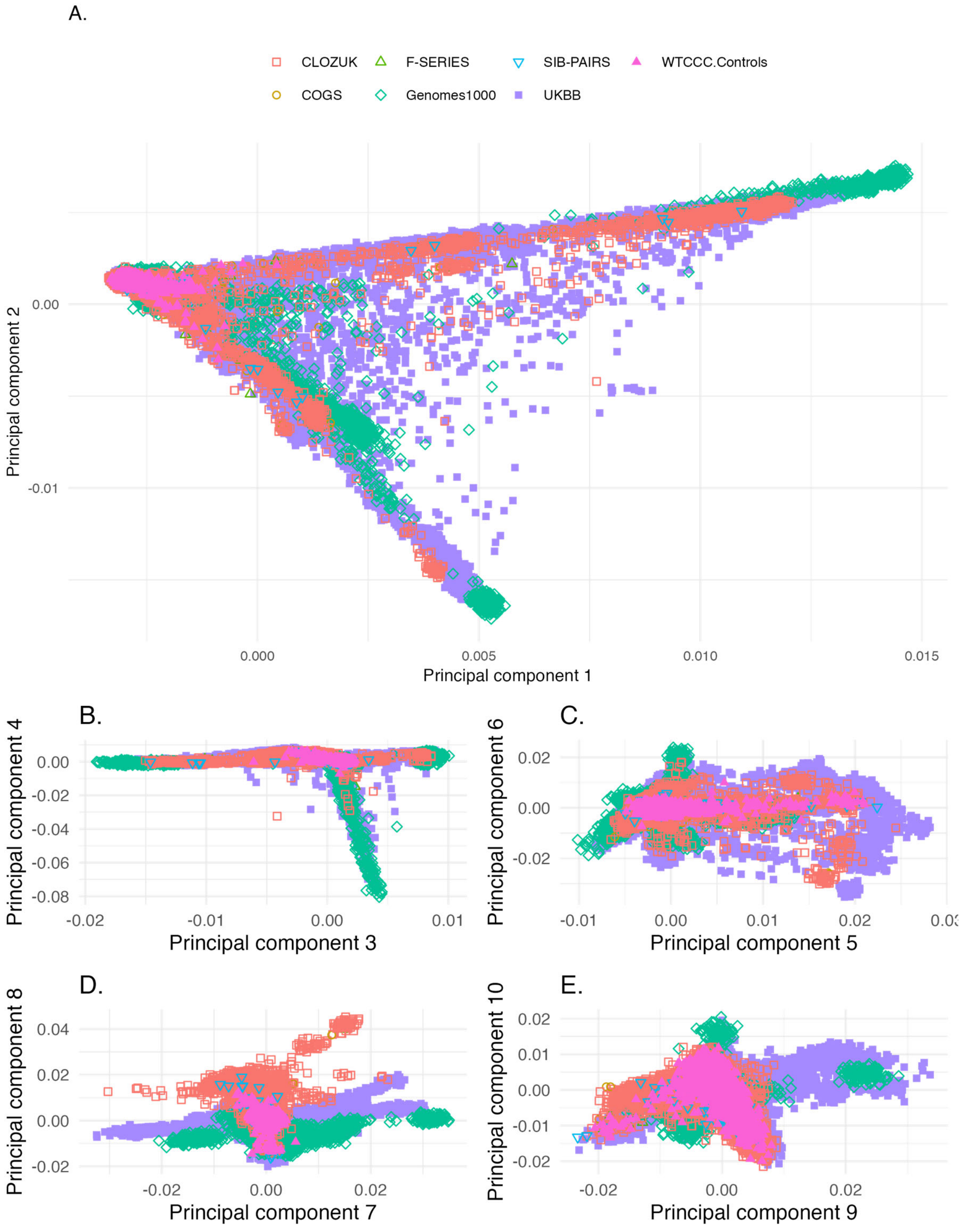

Principal components for each study sample highlighted by colour and shape to check for population stratification due to genotyping array. Plot A displays principal component 1 vs. 2; B principal component 3 vs. 4; C principal component 5 vs. 6; D principal component 7 vs. 8; and E principal component 9 vs. 10.

© 2024 Legge SE et al. *JAMA Psychiatry*.

eFigure 2. PRS Ancestry Adjustment

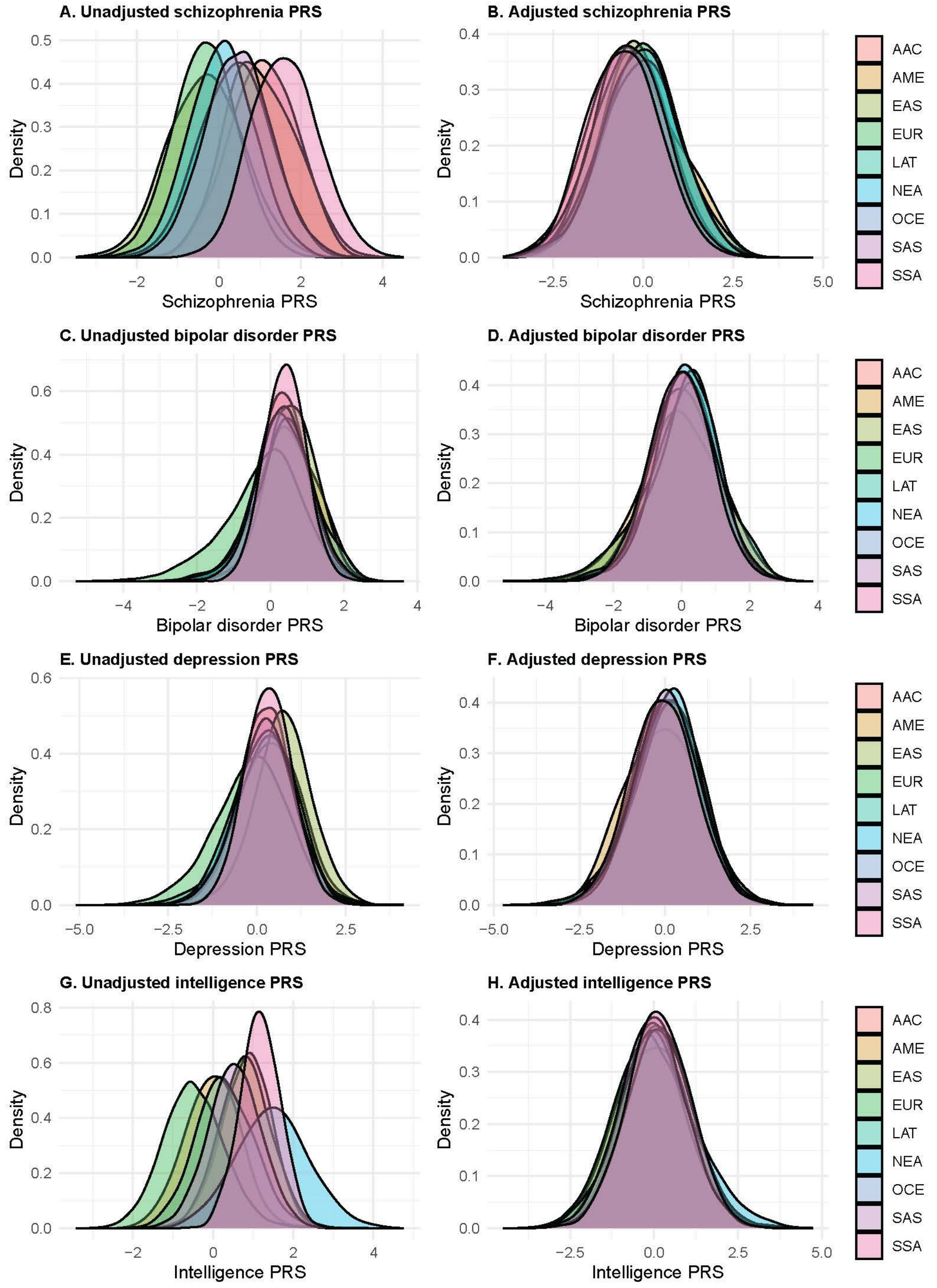

Comparison of the unadjusted and adjusted PRS scores for schizophrenia (A & B), bipolar disorder (C & D), major depressive disorder (E & F), and intelligence (G & G) using the method from Khan et al[10]. Scores are differentiated by the nine biogeographical groupings as described in Supplementary Methods 2[4]. All samples included in the study are plotted. AAC = African American/Afro-Caribbean (admixed); AME = American; EAS = East Asian; EUR = European; LAT = Latino (admixed group); NEA = Near Eastern; OCE = Oceanian; SAS = Central/South Asian; SSA = Sub-Saharan African.

© 2024 Legge SE et al. *JAMA Psychiatry*.

eFigure 3. Biogeographical Genetic Ancestries Groupings

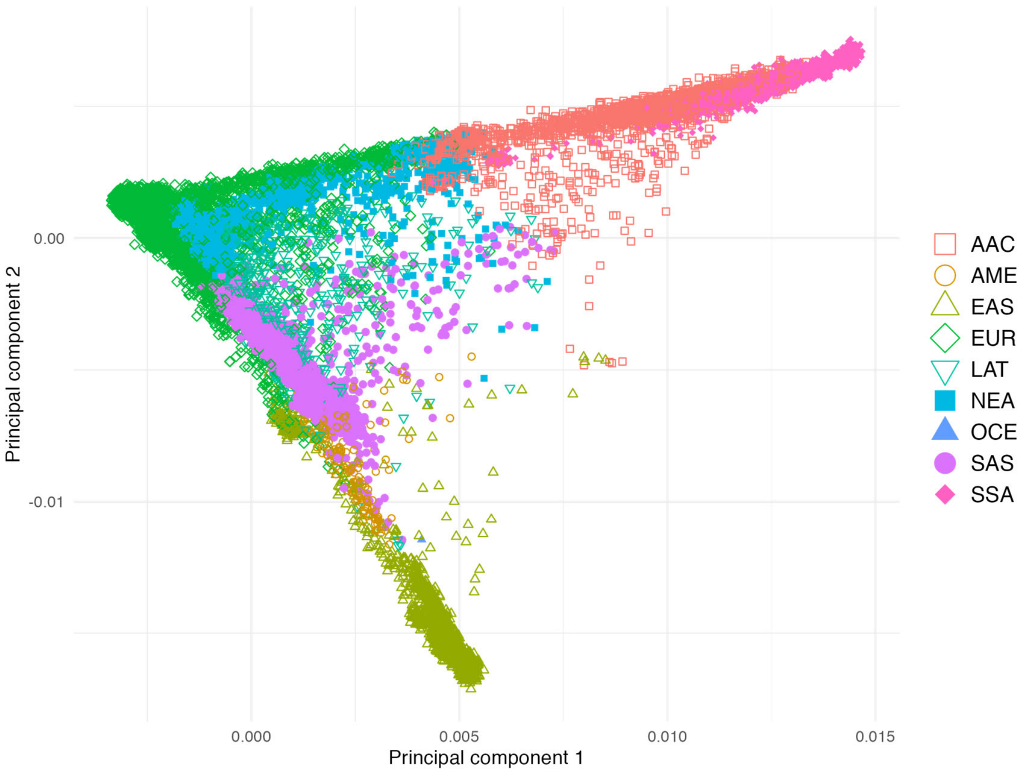

Principal component 1 vs. principal component 2 where the points are differentiated by the nine biogeographical groupings as described in Supplementary Methods[4]. All samples included in the study are plotted. AAC = African American/Afro-Caribbean (admixed); AME = American; EAS = East Asian; EUR = European; LAT = Latino (admixed group); NEA = Near Eastern; OCE = Oceanian; SAS = Central/South Asian; SSA = Sub-Saharan African.

© 2024 Legge SE et al. *JAMA Psychiatry*.

eFigure 4. Manhattan Plot for Schizophrenia in UK Biobank GWAS

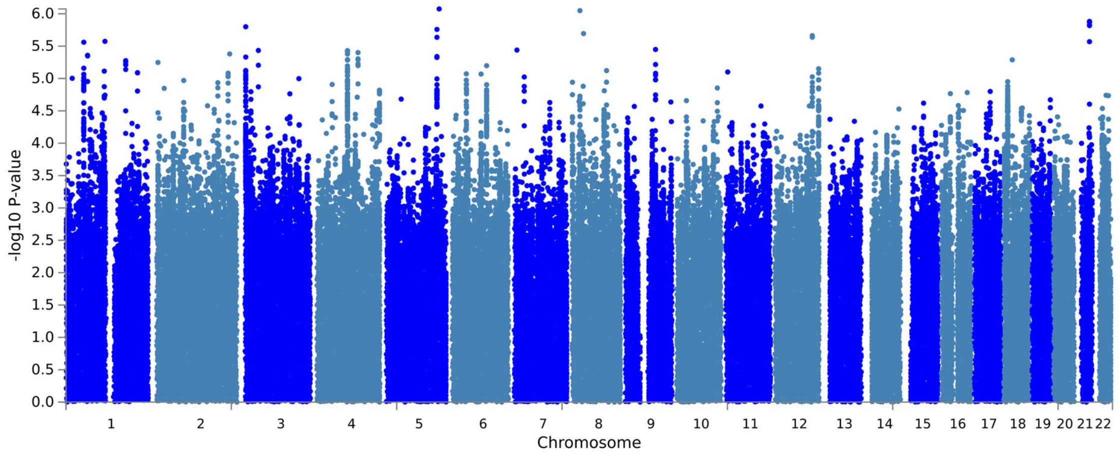

Manhattan Plot of schizophrenia GWAS in UK Biobank. Plot created in FUMA[11].

© 2024 Legge SE et al. *JAMA Psychiatry*.

eFigure 5. QQ Plot for Schizophrenia in UK Biobank GWAS

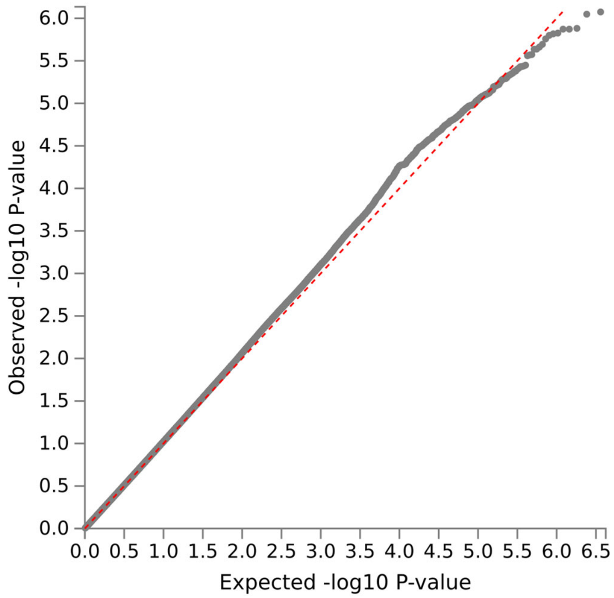

QQ Plot of schizophrenia case/control GWAS in UK Biobank. λ_{GC} = 1.03. Plot created in FUMA[11].

© 2024 Legge SE et al. *JAMA Psychiatry*.

eTable 1. Source of Schizophrenia Diagnosis in UK Biobank

Source of diagnosis	Number
Hospital records only	642
Self-report only	258
Primary care records only	72
Death records only	4
Hospital records plus self-report	440
Hospital records plus primary care records	2
Hospital records plus death records	10
Self-report plus primary care records	1
Self-report plus death records	1
Hospital records plus self-report plus death records	8
Total	1438

Source of diagnoses of schizophrenia in UK Biobank. Total n=1438. Sources included self-report (field ID 20002), ICD-10 F20 medical record diagnosis from hospital admissions (field IDs 41202 and 41204) or death records (field IDs 40001 and 40002), or an equivalent read code from primary care records (field ID 130875).

© 2024 Legge SE et al. *JAMA Psychiatry*.

eTable 2. Number of CNVs per Cohort

CNV	UK Biobank controls			UK Biobank cases			CLOZUK			Clinical samples		
	N carriers	N non-carriers	% CNV Prevalence	N carriers	N non-carriers	% CNV Prevalence	N carriers	N non-carriers	% CNV Prevalence	N carriers	N non-carriers	% CNV Prevalence
1q21.1 del*	100	388271	0.026	0	974	0	22	11828	0.186	0	1073	0
1q21.1 dup*	168	388203	0.043	0	974	0	14	11836	0.118	2	1071	0.186
3q29 del*	6	388365	0.002	2	972	0.205	6	11844	0.051	0	1073	0
15q11.2 del	1536	386835	0.395	4	970	0.411	82	11768	0.692	6	1067	0.559
15q13.3 del*	39	388332	0.010	1	973	0.103	8	11842	0.068	0	1073	0
16p11.2 dup*	121	388250	0.031	3	971	0.308	50	11800	0.422	4	1069	0.373
16p12.1 del	234	388137	0.060	0	974	0	26	11824	0.219	4	1069	0.373
16p13.11 dup	768	387603	0.198	3	971	0.308	44	11806	0.371	4	1069	0.373
NRXN1 del*	157	388214	0.040	1	973	0.103	18	11832	0.152	2	1071	0.186
PWS dup	15	388356	0.004	1	973	0.103	14	11836	0.118	0	1073	0
WBS dup*	11	388360	0.003	1	973	0.103	4	11846	0.034	1	1072	0.093
22q11.2 del*	8	388363	0.002	0	974	0	36	11814	0.304	3	1070	0.280
Any above CNV	3152	385219	0.812	16	958	1.643	324	11526	2.734	26	1047	2.423

Number per schizophrenia associated copy number variation (CNV) observed in each cohort. PWS = Prader-Willi syndrome. WBS = Williams-Beuren syndrome. Analyses restricted to those with European genetic ancestry. Columns represent the CNV, the number of carriers and non-carriers in each cohort, then CNV prevalence. Clinical samples included individuals from CardiffCOGS, Cardiff F-Series, and Cardiff Affected Sib-pairs. *Indicates CNVs associated with schizophrenia with genome-wide significance in the PGC CNV study by Marshall et al, 2017[12].

© 2024 Legge SE et al. *JAMA Psychiatry*.

eTable 3. Phenotype Descriptions

**Phenotype**	**Combined definition**	**UK Biobank**	**CardiffCOGS**	**Cardiff F-Series**	**Cardiff Affected Sib-Pairs**
Male sex	1=Male<br>0=Female	Field ID: 31; sex<br>Not recoded	Not recoded	Not recoded	Not recoded
Age at interview	Age in years	Field ID: 21002; year of birth<br>Not recoded	Not recoded	Not recoded	Not recoded
Year of birth	Year of birth	Field ID: 34; age at recruitment<br>Not recoded	Not recoded	Not recoded	Not recoded
Married/co-habited with a romantic partner	1=Ever married or co-habited<br>0=Never married or co-habited	Field ID: 6141; people in household<br>Recoded: 1 = Ever married or co-habited. All other codes = 0	Self-reported at interview<br>Not recoded	Self-reported at interview<br>Not recoded	Self-reported at interview<br>Not recoded
Number of children (analysed separately in males and females)	Number of live-born children	Field ID: 2405 for males; 2734 for females<br>Not recoded	Self-reported at interview<br>Not recoded	Not available	Self-reported at interview<br>Not recoded
Ever tobacco smoker	1 = Ever regular tobacco smoker<br>0 = Never regular tobacco smoker	Field ID: 20116; smoking status<br>Recoded: 1 and 2 (previous and current) = 1, 0 (never) = 0	Self-reported current smoking and ever smoking status at interview<br>Recoded: Positive rating for either = 1, negative rating for both = 0	Self-reported current smoking and ever smoking status at interview<br>Recoded: Positive rating for either = 1, negative rating for both = 0	Self-reported current smoking and ever smoking status at interview<br>Recoded: Positive rating for either = 1, negative rating for both = 0

© 2024 Legge SE et al. *JAMA Psychiatry*.

Phenotype	Combined definition	UK Biobank	CardiffCOGS	Cardiff F-Series	Cardiff Affected Sib-Pairs
Currently employed	1 = Currently in paid employment. Does not include voluntary work.<br>0 = Not in paid employment.<br>Restricted to individuals of working age (< 65 years).	Field ID: 6142; current employment status<br>Recoded: 1 (in employment) = currently in paid employment. Codes 4 and 5 (Unemployed, unable to work due to disability) = 0, All other codes excluded. Restricted to individuals < 65 years.	Self-reported current occupation at time of interview<br>Recoded into: 1 = all paid occupations, 0 = never worked, unemployed, or not working due to sickness. All other responses coded as missing and restricted to individuals < 65 years.	Not available	Self-reported employment since onset of schizophrenia<br>1 = Yes<br>0 = No<br>Recoded to remove individuals > 65 years.
Highschool qualification (GCSEs)	1 = Achieved GCSEs or equivalent<br>0 = Have not achieved GCSEs or equivalent	Field ID: 6138; qualifications<br>Recoded: codes 1,2,3 = 1, remaining codes = 0	Self-reported highest educational qualification<br>Recoded: GCSEs and equivalent or above = 1, else = 0	Self-reported highest educational qualification<br>Recoded: GCSEs and equivalent or above = 1, else = 0	Self-reported highest educational qualification<br>Recoded: GCSEs and equivalent or above = 1, else = 0
Degree	1 = Achieved university degree<br>0 = Have not achieved university degree	Field ID: 6138; qualifications<br>Recoded: codes 1 = 1, remaining codes = 0	Self-reported highest educational qualification<br>Recoded: Degree or above = 1, else = 0	Self-reported highest educational qualification<br>Recoded: Degree or above = 1, else = 0	Self-reported highest educational qualification<br>Recoded: Degree or above = 1, else = 0
Cognitive ability	Cognitive ability standardised in each sample against unaffected controls	Field ID: 20016; fluid intelligence<br>Recoded: Standardised against unaffected controls	MATRICS composite score standardised against unaffected controls<br>Not recoded	Not available	Not available

© 2024 Legge SE et al. *JAMA Psychiatry*.

Phenotype	Combined definition	UK Biobank	CardiffCOGS	Cardiff F-Series	Cardiff Affected Sib-Pairs
ICD-10 Depression	1 = Present<br>0 = Absent	Field ID: 130895, 130897; first occurrences fields for ICD-10 depressive episode and MDD.<br>Recoded: Any codes 20:51 from either field = 1, no codes in either field = 0	Semi-structured clinical interview (SCAN) completed with participants and confirmed in medical case records where available<br>Not recoded	Semi-structured clinical interview (SCAN) completed with participants and confirmed in medical case records where available<br>Not recoded	Semi-structured clinical interview (SCAN) completed with participants and confirmed in medical case records where available<br>Not recoded
Epilepsy	1 = Present<br>0 = Absent	Field ID: 131049; first occurrences field for ICD-10 epilepsy.<br>Recoded: Any codes 20:51 = 1, else = 0	Self-reported at interview<br>Not recoded	Not available	Not available
Type 2 diabetes	1 = Present<br>0 = Absent	Field ID: 130709; first occurrences field for ICD-10 type 2 diabetes.<br>Recoded: Any codes 20:51 = 1, else = 0	Self-reported at interview<br>Not recoded	Not available	Not available
Heart disease	1 = Present<br>0 = Absent	Field ID: 131297; first occurrences field for ICD-10 heart disease.<br>Recoded: Any codes 20:51 = 1, else = 0	Self-reported at interview<br>Not recoded	Not available	Not available

© 2024 Legge SE et al. *JAMA Psychiatry*.

Phenotype	Combined definition	UK Biobank	CardiffCOGS	Cardiff F-Series	Cardiff Affected Sib-Pairs
Onset of psychosis	Age in years at onset of psychotic symptoms	Field ID: 20009 when 20002 indicated schizophrenia; self-reported age of onset of when disorder started.<br>Not recoded	Self-reported at interview and confirmed in medical case records where available<br>Not recoded	Self-reported at interview and confirmed in medical case records where available<br>Not recoded	Self-reported at interview and confirmed in medical case records where available<br>Not recoded

Definitions of phenotypes used and recoding conducted to combine data from the contributing samples. Columns represent the phenotype (Phenotype), combined definition and coding used in analyses (Combined definition), then detail the original phenotype and any recoding of said phenotype for each sample (UK Biobank, CardiffCOGS, Cardiff F-Series, Cardiff Affected Sib-Pairs). Onset of psychosis (n=638) and cognitive ability (n=451) were only available for a subset of UK Biobank schizophrenia cases.

© 2024 Legge SE et al. *JAMA Psychiatry*.

eTable 4. Genetic Correlations With Schizophrenia in UK Biobank

	r_g with UK Biobank schizophrenia			r_g with PGC schizophrenia			Comparison	
	r_g	r_g SE	r_g p-value	r_g	r_g SE	r_g p-value	X^2	X^2 p-value
PGC3 schizophrenia[13]	0.98	0.18	3.55×10^{-8}	-	-	-	-	-
Bipolar disorder[14]	0.73	0.14	1.15×10^{-7}	0.68	0.02	0	0.125	0.724
MDD[15]	0.34	0.08	1.93×10^{-5}	0.32	0.02	1.25×10^{-51}	0.059	0.801
ADHD[16]	0.12	0.09	0.173	0.16	0.03	1.16×10^{-7}	0.178	0.673
ASD[17]	0.17	0.10	0.097	0.25	0.04	1.66×10^{-12}	0.552	0.458
Anorexia nervosa[18]	0.32	0.11	3.01×10^{-3}	0.22	0.03	2.45×10^{-15}	0.769	0.380
Cannabis use disorder[19]	0.18	0.12	0.121	0.34	0.04	3.83×10^{-22}	1.6	0.206
Alcohol use disorder[20]	0.46	0.17	7.80×10^{-3}	0.36	0.06	1.41×10^{-10}	0.308	0.579
Intelligence[21]	-0.14	0.06	0.030	-0.20	0.02	9.43×10^{-23}	0.9	0.343

Genetic correlations between the schizophrenia GWAS in UK Biobank and other neuropsychiatric disorders, PGC schizophrenia GWAS[13] and other neuropsychiatric disorders, and the chi-square statistic (X^2) to test for the difference between the two. Columns represent the genetic correlation (r_g), standard error (SE) and p-value.

© 2024 Legge SE et al. *JAMA Psychiatry*.

eTable 5. Polygenic Risk Score Comparisons Between Cohorts

		Schizophrenia PRS		Bipolar disorder PRS		Depression PRS		Intelligence PRS	
**Test sample**	**Comparator sample**	**OR (95% CI)**	**P**	**OR (95% CI)**	**P**	**OR (95% CI)**	**P**	**OR (95% CI)**	**P**
Case-control									
UK Biobank cases	UK Biobank controls	1.69 (1.59,1.78)	$3.79x10^{-71}$	1.20 (1.13,1.27)	$4.29x10^{-10}$	1.06 (1.00,1.12)	0.045	0.89 (0.85,0.94)	$3.34x10^{-5}$
Case-case									
UK Biobank cases	CardiffCOGS	0.94 (0.85,1.04)	0.205	0.99 (0.91,1.09)	0.909	0.94 (0.85,1.04)	0.228	1.12 (1.02,1.22)	0.019
UK Biobank cases	CLOZUK	0.81 (0.76,0.85)	$2.59x10^{-13}$	0.98 (0.93,1.04)	0.521	0.95 (0.90,1.00)	0.069	1.06 (1.00,1.11)	0.042
UK Biobank cases	Cardiff F-Series	0.75 (0.67,0.85)	$4.25x10^{-6}$	0.99 (0.89,1.10)	0.817	0.94 (0.84,1.06)	0.298	0.94 (0.84,1.05)	0.255
UK Biobank cases	Cardiff Affected Sib-Pairs	0.82 (0.69,0.99)	0.036	0.93 (0.78,1.10)	0.397	0.97 (0.81,1.17)	0.769	0.93 (0.78,1.10)	0.373

Schizophrenia, bipolar disorder, major depressive disorder (Depression), and intelligence polygenic risk score (PRS) comparisons between UK Biobank cases (Test sample) and the other samples (listed in Comparator sample). Odds ratios refer to UK Biobank cases; values > 1 indicate greater scores in UK Biobank schizophrenia cases and values < 1 indicate lower values.

© 2024 Legge SE et al. *JAMA Psychiatry*.

eTable 6. PRS Analyses in Individuals of European Genetic Ancestry

		Schizophrenia PRS		Bipolar disorder PRS		Depression PRS		Intelligence PRS	
**OR sample**	**Ref sample**	**OR (95% CI)**	**P**	**OR (95% CI)**	**P**	**OR (95% CI)**	**P**	**OR (95% CI)**	**P**
Case-control									
UK Biobank cases	UK Biobank controls	1.86 (1.73,2.00)	1.99×10^{-65}	1.39 (1.30,1.49)	6.85×10^{-22}	1.15 (1.08,1.23)	1.80×10^{-5}	0.97 (0.90,1.04)	0.375
Case-case									
UK Biobank cases	CardiffCOGS	0.97 (0.88,1.10)	0.811	0.97 (0.87,1.08)	0.621	0.91 (0.82,1.01)	0.092	1.13 (1.01,1.26)	0.040
UK Biobank cases	CLOZUK	0.78 (0.73,0.83)	2.92×10^{-13}	0.99 (0.92,1.05)	0.656	0.93 (0.88,0.99)	0.029	1.09 (1.02,1.17)	0.015
UK Biobank cases	Cardiff F-Series	0.78 (0.68,0.89)	2.26×10^{-4}	0.98 (0.87,1.11)	0.765	0.95 (0.84,1.08)	0.298	0.95 (0.83,1.08)	0.415
UK Biobank cases	Cardiff Affected Sib-Pairs	0.85 (0.70,1.04)	0.119	0.92 (0.76,1.11)	0.420	0.95 (0.78,1.14)	0.574	0.92 (0.74,1.13)	0.419

Unadjusted PRS-CS polygenic risk score analyses repeated only in individuals of European genetic ancestry covarying for principal components. Columns represent the reference sample (Ref sample), the sample the odds ratio is in reference to (OR sample), odds ratio and 95% confidence intervals (OR, 95% CI), and p-value (P) for schizophrenia, bipolar disorder, major depressive disorder and intelligence PRS.

© 2024 Legge SE et al. *JAMA Psychiatry*.

eTable 7. Phenotypic Characteristics per Cohort

Phenotype	UK Biobank Controls Total n=499421	UK Biobank cases Total n=1438	CardiffCOGS Total n=767	Cardiff F-Series Total n=648	Cardiff Affected Sib-Pairs Total n=381
Male sex	0.46 (227537/499421)	0.62 (888/1438)	0.70 (540/767)	0.75 (453/648)	0.69 (262/381)
Married/co-habiting	0.89 (43819/405661)	0.62 (367/589)	0.48 (361/746)	0.42 (262/636)	0.42 (152/365)
Currently employed	0.91 (27215/305210)	0.24 (228/944)	0.10 (67/609)	-	0.08 (17/212)
GCSEs	0.83 (84954/494834)	0.85 (1189/1405)	0.64 (474/745)	0.53 (315/595)	0.38 (98/257)
Degree	0.33 (160231/489381)	0.26 (358/1381)	0.13 (98/745)	0.10 (58/595)	0.04 (9/257)
Ever tobacco smoker	0.45 (224354/496514)	0.60 (840/1407)	0.78 (538/753)	0.69 (418/605)	0.77 (147/191)
ICD-10 depression	0.12 (58015/499421)	0.48 (688/1438)	0.58 (398/685)	0.17 (105/628)	0.22 (79/364)
Epilepsy	0.02 (8148/499421)	0.07 (107/1438)	0.09 (51/665)	-	-
Type 2 diabetes	0.08 (41516/457905)	0.22 (321/1438)	0.14 (90/666)	-	-
Heart disease	0.12 (59926/499421)	0.20 (286/1438)	0.07 (31/664)	-	-
Age at interview in years	56.54 (sd= 8.09)	54.70 (sd=8.31)	42.90 (sd=12.29)	41.97 (sd=12.04)	41.49 (sd=12.57)
Year of birth	1952 (sd=8.11)	1953 (sd=8.33)	1968 (sd=12.68)	1957 (sd=14.16)	1953 (sd=12.94)
Number of children (males)	1.82 (sd=1.31)	1.13 (sd=2.89)	0.54 (sd=1.11)	-	0.42 (sd=1.06)
Number of children (females)	1.82 (sd=1.20)	1.43 (sd=1.51)	1.12 (sd=1.65)	-	1.52 (sd=1.68)
Cognitive ability	0.00 (sd=1.00)	-0.57 (sd=0.98)	-2.40 (sd=1.34)	-	-
Onset of psychosis in years	-	31.48 (sd=10.97)	24.32 (sd=8.70)	23.83 (sd=8.28)	23.81 (sd=7.29)

Phenotype characteristics for each cohort. Values are unadjusted for age and sex. Phenotype definitions are detailed in Supplementary Table 2. Onset of psychosis (n=638) and cognitive ability (n=451) were only available for a subset of UK Biobank schizophrenia cases.

© 2024 Legge SE et al. *JAMA Psychiatry*.

eTable 8. Genetic Ancestry Differences Between Case Cohorts

	UK Biobank SZ		CLOZUK	
**Biogeographic ancestry**	**n**	**Proportion**	**n**	**Proportion**
AAC	50	0.037	605	0.043
EAS	9	0.007	100	0.007
EUR	1172	0.861	12342	0.874
NEA	17	0.012	288	0.020
SAS	55	0.040	530	0.038
SSA	57	0.042	245	0.017
All	1361	1.000	14117	1.000

Number of individuals from UK Biobank with schizophrenia and CLOZUK from each biogeographic ancestry group. Groups with > 5 individuals in UK Biobank schizophrenia cases are presented. The biogeographical groupings are described in Supplementary Methods 2[4]. AAC = African American/Afro-Caribbean (admixed); EAS = East Asian; EUR = European; NEA = Near Eastern; SAS = Central/South Asian; SSA = Sub-Saharan African.

© 2024 Legge SE et al. *JAMA Psychiatry*.

eTable 9. Self-Reported Ethnicity and Schizophrenia in UK Biobank

	UK Biobank SZ		UK Biobank controls	
**Biogeographic ancestry**	**n**	**Proportion**	**n**	**Proportion**
Asian or Asian British	46	0.032	9814	0.020
Black or Black British	89	0.062	7935	0.016
Chinese	4	0.003	1565	0.003
Mixed	32	0.022	2903	0.006
White	1208	0.847	469969	0.943
Other or unknown	47	0.033	6353	0.013
All	1426	1.000	498539	1.000

Self-reported ethnicity of individuals in UK Biobank with schizophrenia (UK Biobank SZ) and without a psychotic disorder (UK Biobank controls).

© 2024 Legge SE et al. *JAMA Psychiatry*.

eReferences.

1. Legge SE, Pardinas AF, Helthuis M, et al. A genome-wide association study in individuals of African ancestry reveals the importance of the Duffy-null genotype in the assessment of clozapine-related neutropenia. *Mol Psychiatry.* 2019;24(3):328-337.
2. Leutenegger AL, Sahbatou M, Gazal S, Cann H, Génin E. Consanguinity around the world: what do the genomic data of the HGDP-CEPH diversity panel tell us? *Eur J Hum Genet.* 2011;19(5):583-587.
3. Li JZ, Absher DM, Tang H, et al. Worldwide human relationships inferred from genome-wide patterns of variation. *Science.* 2008;319(5866):1100-1104.
4. Huddart R, Fohner AE, Whirl-Carrillo M, et al. Standardized Biogeographic Grouping System for Annotating Populations in Pharmacogenetic Research. *Clin Pharmacol Ther.* 2019;105(5):1256-1262.
5. Zheng X, Levine D, Shen J, Gogarten SM, Laurie C, Weir BS. A high-performance computing toolset for relatedness and principal component analysis of SNP data. *Bioinformatics.* 2012;28(24):3326-3328.
6. Galinsky KJ, Bhatia G, Loh PR, et al. Fast Principal-Component Analysis Reveals Convergent Evolution of ADH1B in Europe and East Asia. *Am J Hum Genet.* 2016;98(3):456-472.
7. Brodersen KH, Ong CS, Stephan KE, Buhmann JM. The Balanced Accuracy and Its Posterior Distribution. Paper presented at: 2010 20th International Conference on Pattern Recognition; 23-26 Aug. 2010, 2010.
8. Legge SE, Jones HJ, Kendall KM, et al. Association of Genetic Liability to Psychotic Experiences With Neuropsychotic Disorders and Traits. *JAMA Psychiatry.* 2019;76(12):1256-1265.
9. Laursen TM, Agerbo E, Pedersen CB. Bipolar disorder, schizoaffective disorder, and schizophrenia overlap: a new comorbidity index. *J Clin Psychiatry.* 2009;70(10):1432-1438.
10. Khan A, Turchin MC, Patki A, et al. Genome-wide polygenic score to predict chronic kidney disease across ancestries. *Nat Med.* 2022;28(7):1412-1420.
11. Watanabe K, Taskesen E, van Bochoven A, Posthuma D. Functional mapping and annotation of genetic associations with FUMA. *Nat Commun.* 2017;8(1):1826.
12. Marshall CR, Howrigan DP, Merico D, et al. Contribution of copy number variants to schizophrenia from a genome-wide study of 41,321 subjects. *Nat Genet.* 2017;49(1):27-35.
13. Trubetskoy V, Pardiñas AF, Qi T, et al. Mapping genomic loci implicates genes and synaptic biology in schizophrenia. *Nature.* 2022;604(7906):502-508.
14. Mullins N, Forstner AJ, O'Connell KS, et al. Genome-wide association study of more than 40,000 bipolar disorder cases provides new insights into the underlying biology. *Nat Genet.* 2021;53(6):817-829.
15. Howard DM, Adams MJ, Clarke TK, et al. Genome-wide meta-analysis of depression identifies 102 independent variants and highlights the importance of the prefrontal brain regions. *Nat Neurosci.* 2019;22(3):343-352.
16. Demontis D, Walters RK, Martin J, et al. Discovery of the first genome-wide significant risk loci for attention deficit/hyperactivity disorder. *Nat Genet.* 2019;51(1):63-75.
17. Grove J, Ripke S, Als TD, et al. Identification of common genetic risk variants for autism spectrum disorder. *Nat Genet.* 2019;51(3):431-444.
18. Watson HJ, Yilmaz Z, Thornton LM, et al. Genome-wide association study identifies eight risk loci and implicates metabo-psychiatric origins for anorexia nervosa. *Nat Genet.* 2019;51(8):1207-1214.

© 2024 Legge SE et al. *JAMA Psychiatry*.

19. Johnson EC, Demontis D, Thorgeirsson TE, et al. A large-scale genome-wide association study meta-analysis of cannabis use disorder. *Lancet Psychiatry.* 2020;7(12):1032-1045.
20. Walters RK, Polimanti R, Johnson EC, et al. Transancestral GWAS of alcohol dependence reveals common genetic underpinnings with psychiatric disorders. *Nat Neurosci.* 2018;21(12):1656-1669.
21. Savage JE, Jansen PR, Stringer S, et al. Genome-wide association meta-analysis in 269,867 individuals identifies new genetic and functional links to intelligence. *Nat Genet.* 2018;50(7):912-919.

© 2024 Legge SE et al. *JAMA Psychiatry*.
