# Supplementary material for: Genetic and Phenotypic Features of Schizophrenia in the UK Biobank
Source: JAMA Psychiatry. 2024 Mar 27;81(7):681–90. doi: 10.1001/jamapsychiatry.2024.0200 (PMC10974692; doi:10.1001/jamapsychiatry.2024.0200)
Supplement: Supplement 2. — Data Sharing Statement [file jamapsychiatry-e240200-s002.pdf]

Data Sharing Statement

Legge. Genetic and Phenotypic Features of Schizophrenia in the UK Biobank. *JAMA Psychiatry*. Published March 27, 2024. doi:10.1001/jamapsychiatry.2024.0200

Data
Data available: No

Additional Information
Explanation for why data not available: UK Biobank data can be obtained upon application from https://www.ukbiobank.ac.uk/enable-your-research. UK Biobank schizophrenia GWAS summary statistics can be downloaded from https://walters.psycm.cf.ac.uk/. The de-duplicated PGC summary stats for schizophrenia are available on the PGC website https://figshare.com/articles/dataset/scz2022/19426775. All code will be made available upon request. Data from the clinically-ascertained cohorts can not be made publicly available due to restrictions in ethical approvals.
